# Extracellular Transglutaminase 2 Is Catalytically Inactive, but Is Transiently Activated upon Tissue Injury

**DOI:** 10.1371/journal.pone.0001861

**Published:** 2008-03-26

**Authors:** Matthew Siegel, Pavel Strnad, R. Edward Watts, Kihang Choi, Bana Jabri, M. Bishr Omary, Chaitan Khosla

**Affiliations:** 1 Department of Chemical Engineering, Stanford University, Stanford, California, United States of America; 2 Department of Medicine, VA Palo Alto Health Care System, Palo Alto, California, United States of America; 3 Department of Chemistry, Stanford University, Stanford, California, United States of America; 4 Department of Pathology, Medicine and Pediatrics, University of Chicago, Chicago, Illinois, United States of America; 5 Department of Biochemistry, Stanford University, Stanford, California, United States of America; Cairo University, Egypt

## Abstract

Transglutaminase 2 (TG2) is a multifunctional mammalian protein with transamidase and signaling properties. Using selective TG2 inhibitors and tagged nucleophilic amine substrates, we show that the majority of extracellular TG2 is inactive under normal physiological conditions in cell culture and *in vivo*. However, abundant TG2 activity was detected around the wound in a standard cultured fibroblast scratch assay. To demonstrate wounding-induced activation of TG2 *in vivo*, the toll-like receptor 3 ligand, polyinosinic-polycytidylic acid (poly(I:C)), was injected in mice to trigger small intestinal injury. Although no TG2 activity was detected in vehicle-treated mice, acute poly(I:C) injury resulted in rapid TG2 activation in the small intestinal mucosa. Our findings provide a new basis for understanding the role of TG2 in physiology and disease.

## Introduction

Transglutaminase 2 (TG2, tissue transglutaminase, transglutaminase C, G_αh_) is a multifunctional protein found in most mammalian tissues capable of enzymatic, cell adhesion, cell signaling, and G-protein activities [1]–[4]. A ubiquitous protein, its function is presumably dictated by its cellular localization, interaction with other proteins and binding to cofactors [1]–[3]. In the presence of calcium and the absence of GTP or GDP, TG2 can act as an enzyme capable of post-translationally modifying proteins through transamidation or deamidation. As a transamidase, TG2 catalyzes the formation of an isopeptide bond (intermolecular ε-(γ-glutamyl) lysine crosslink) between a selected glutamine residue on one substrate and a lysine residue on a second substrate. Alternatively, certain small molecule amines can also be attached onto glutamine side-chains of proteins by TG2. In the absence of adequate concentrations of any suitable amine substrate, TG2 deamidates the targeted glutamine side chain, resulting in its conversion to glutamate [2], [3], [5].

The enzymatic activity of TG2 is of pharmacological interest because it is believed to contribute to the pathogenesis of diseases such as celiac sprue [6], neurodegenerative disorders [7], type 2 diabetes [8], and certain cancers [9]–[11]. For example, in celiac sprue gluten peptides resistant to proteolysis by gastro-pancreatic proteases [12] are deamidated by TG2 in the small intestine of patients [13]. The negative charge introduced by peptide deamidation increases the affinity of these gluten peptides for the disease associated HLA-DQ2 protein [14] thereby triggering an inflammatory T cell response that ultimately leads to the destruction of small intestinal architecture [15]. Furthermore, an anti-TG2 antibody response is mounted in patients on a gluten-containing diet but is silenced when gluten is excluded from the diet suggesting the close interaction of gluten peptides with TG2 [15].

Due to the therapeutic potential of inhibiting TG2 enzyme activity, many classes of TG2 irreversible inhibitors that form stable covalent bonds with the TG2 active site cysteine have been synthesized [16], [17]. Among these inhibitors, 3-bromo-4,5-dihydroisoxazoles are among the most well-studied from a structure-activity and biological viewpoint [11], [18]–[20]. These inhibitors are also useful chemical probes for assessing the enzymatic activity of TG2 in biological systems. Most previous attempts to study TG2 activity in mammalian tissues involved tissue homogenization and lysis, followed by a crosslinking activity assay of the lysate using buffers with millimolar levels of calcium [21]. Not only does this method result in a loss of the tissue localization of TG2, but the presence of high calcium concentrations in the buffer leads to activation of enzymatically latent TG2 (vide infra). Therefore, although this method is appropriate for quantifying the abundance of TG2 *protein* in tissue samples, it does not report on TG2 enzyme activity *in vivo*. Alternate methods for assaying *in situ* TG2 activity involve the incubation of histological sections with labeled amine substrates such as monodansyl cadaverine or 5-biotinamidopentylamine [22], [23]. Again, these assays entail incubation of tissue samples in calcium buffers and are often preceded by fixation or comparable perturbation of the tissue.

In this study, we probed the activity of TG2 in intact biological systems. A variety of active site-directed inhibitors and tagged nucleophilic amine substrates (5-biotinamidopentylamine, fluorescein cadaverine, and ^14^C-putrescine) of TG2 were synthesized and evaluated. While previous studies suggest the enzymatic latency of intracellular TG2 in cell culture [24], [25], our data suggest that the majority of extracellular TG2 is also inactive despite an environment conducive to enzyme activation. On the other hand, TG2 could be enzymatically activated and inhibited in a standard cultured fibroblast wounding model. In an attempt to demonstrate wounding-induced activation of latent TG2 *in vivo*, the polyinosinic-polycytidylic acid (poly(I:C)) small intestinal injury model [26] was used to examine *in vivo* TG2 activity. In accord with the fibroblast culture assay and consistent with a role for active TG2 following tissue injury, small intestinal TG2 was enzymatically activated in the acute poly(I:C) injury model. Our results provide a mechanistic framework to understand the catalytic and non-catalytic functions of mammalian TG2 in physiology and disease.

## Results

### Cellular TG2 is not inhibited by dihydroisoxazole inhibitors

In order to determine if 3-bromo-4,5-dihydroisoxazole irreversible inhibitors are able to covalently bind cellular TG2, 100 µM compound **1** was diluted into culture media and incubated at 37°C for one hour with WI-38 fibroblasts and MDA-MB-231 cells, two cell lines previously shown to express abundant TG2 protein [27]–[30]. To evaluate the level of inhibition of TG2 activity, the cells were subsequently washed, lysed by sonication, divided into four aliquots of lysate (see below), incubated for 30 minutes at ambient temperature, and diluted into a calcium-rich activity assay buffer to measure TG2 catalyzed putrescine incorporation into dimethyl casein.

**Figure 1:**
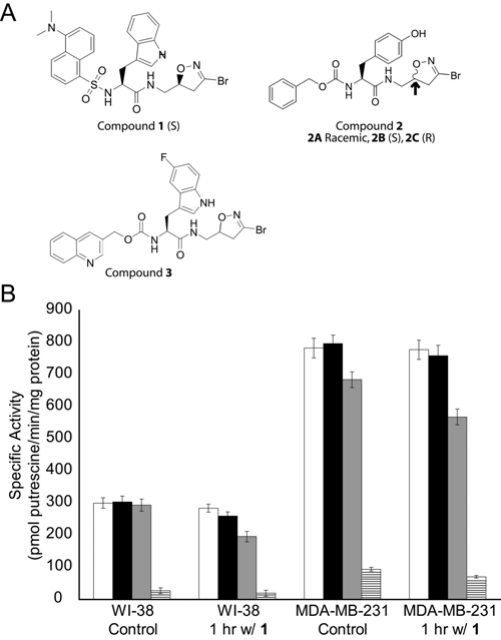
Dihydroisoxazole inhibitors do not inhibit TG2 in intact cells. (A) Chemical structures of the 3-bromo-4,5-dihydroisoxazole inhibitors used in these studies [18], [19]. In some experiments described herein, the stereoisomers of compound 2A (compounds 2B and 2C) at the C-5 carbon of the dihydroisoxazole ring (see arrow) were used. The (S)-isomer (compound 2B) irreversibly inhibits recombinant human TG2 while the (R)-isomer (compound 2C) does not [19]. (B) WI-38 fibroblasts and MDA-MB-231 cells were incubated with or without 100 µM inhibitor 1 for 1 hour. Cells were washed with PBS and detached from the culture plate using 10 mM EDTA in PBS for MDA-MB-231 cells or using trypsin-EDTA in PBS for WI-38 cells. After lysing the cells via sonication, the lysate was split into four equal aliquots and incubated with □ 1% DMSO, ▪ 1% DMSO+100 µM 1, ▪ 1% DMSO+5 mM CaCl_2_, or ——□ 1% DMSO+100 µM 1+5 mM CaCl_2_ for 30 minutes at room temperature. TG2-catalyzed putrescine incorporation into dimethyl casein in a calcium-rich reaction buffer was then used to quantify the amount of *ex vivo* TG2 activity in each fraction. Activities were normalized by total protein concentrations.

The first aliquot contained vehicle (1% DMSO) and was analyzed to determine the level of TG2 inhibition that occurred during cell culture. As shown in Figure 1B, no significant inhibition was observed indicating the enzymatic latency of the majority of cellular TG2. The second aliquot of lysate, into which 100 µM inhibitor **1** in 1% DMSO was added, also did not result in inhibition of TG2 activity implying that inhibitor **1** was unable to irreversibly bind TG2 after cell lysis. The third aliquot was a control that contained 1% DMSO with 5 mM CaCl_2_. The level of TG2 activity decreased slightly in these samples possibly due residual inhibitor **1** left over from incomplete washing of the cells and/or the increased susceptibility of TG2 to proteolysis in the presence of calcium [31], [32]. The fourth aliquot of cell lysate received 100 µM inhibitor **1** and 5 mM CaCl_2_ in 1% DMSO and resulted in nearly complete inhibition of TG2 activity (Figure 1B). In summary, these results indicate that (i) the majority of cellular TG2 cannot be inhibited in intact cells; (ii) dihydroisoxazole inhibitor **1** potently inhibits cell lysate TG2 activity in the presence of calcium; and (iii) calcium in the putrescine incorporation assay buffer results in activation of previously latent TG2, suggesting that this assay is not a reliable indicator of *in vivo* TG2 activity. Rather, it is useful in assessing total TG2 protein content or total *potential* TG2 activity (which we will hereafter refer to as “*ex vivo* TG2 activity”).

### The majority of small intestinal and liver TG2 cannot be inhibited under normal physiological conditions

**Figure 2:**
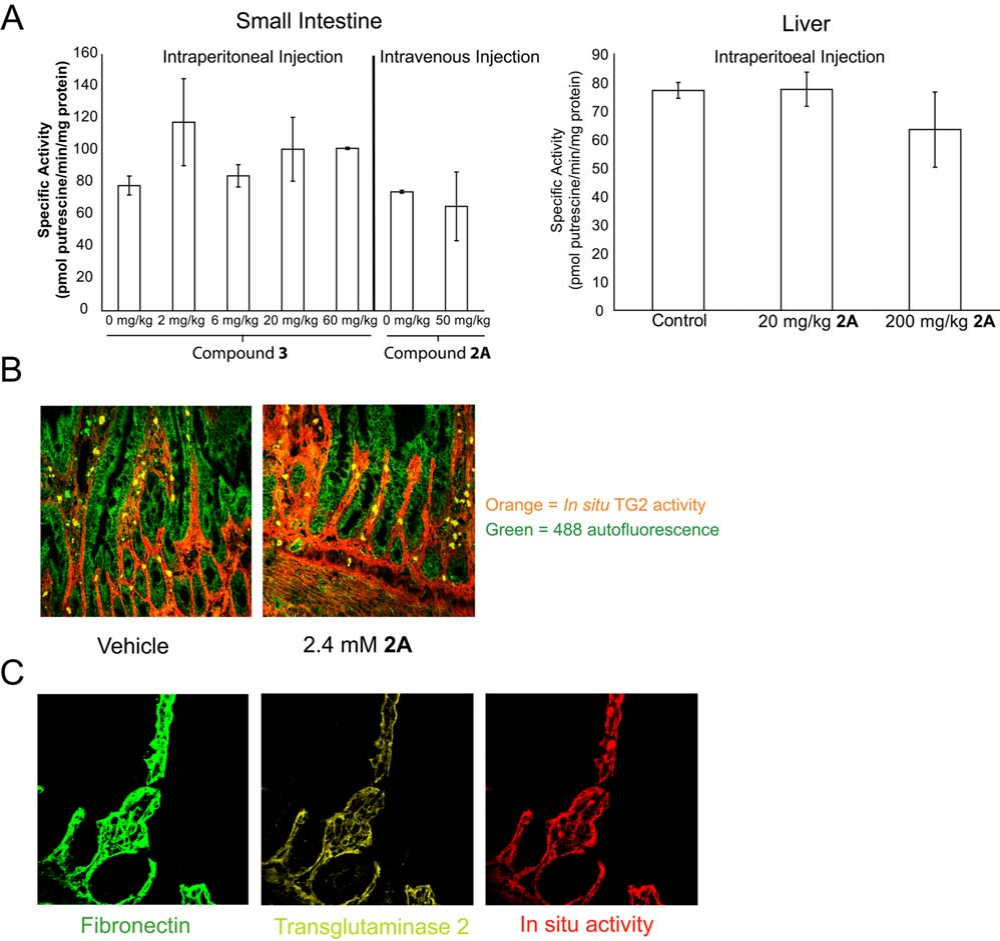
No detectable inhibition of TG2 in the small intestine or liver of mice or rats treated with TG2 inhibitors. (A) Small intestine: Mice were intraperitoneally (IP) injected with vehicle, 2 mg/kg 3, 6 mg/kg 3, 20 mg/kg 3, or 60 mg/kg 3 or intravenously injected with vehicle or 50 mg/kg 2A before being sacrificed 1 hour post-injection. Small intestinal tissue was flushed with PBS before freezing on dry ice. Liver: Mice were IP injected with vehicle, 20 mg/kg 2A, or 200 mg/kg 2A and sacrificed 1 hour post-injection. TG2 activity in the tissue lysates was determined using the putrescine incorporation assay. (Average of two mice. P>0.1 for all doses except 60 mg/kg 3) (B) *Ex vivo* TG2 activity (orange) was measured using 5-BP in tissue sections from the small intestine of rats perfused with vehicle or 2.4 mM 2A. Tissue autofluorescence using the 488 nm laser was collected to help orient and visualize the villous structure of the intestine. (400×) (C) Staining of rat small intestinal tissue for fibronectin (green), TG2 (yellow), and *ex vivo* TG2 activity (red) revealed nearly perfect overlap of all three stains. (400×)

Because TG2 is highly expressed in the liver and small intestine, we selected these two organs as targets for TG2 inhibition by injecting mice with two previously described active-site directed irreversible inhibitors from the 3-bromo-4,5-dihydroisoxazole family of compounds (compounds **2A** and **3**, Figure 1A) [18], [19]. Following animal sacrifice, the organs were harvested, homogenized and assayed for *ex vivo* TG2 activity (i.e., TG2 activated *ex vivo* by calcium in the assay buffer, see Figure 1B) using putrescine incorporation. Initially we observed a dose-dependent decrease in *ex vivo* small intestinal TG2 activity after injecting dihydroisoxazole inhibitors dissolved in 50% DMSO/PBS although no consistent inhibition was observed in the liver (liver, Figure 2A; small intestine, [18]). However, in subsequent experiments, no TG2 inhibition was observed in the small intestine following a thorough rinse of the small intestinal lumen with PBS prior to tissue harvesting (Figure 2A). This suggests that the inhibition observed without intestinal washing was most likely due to TG2 inactivation by residual amounts of inhibitor that was carried into the tissue homogenization and lysis steps. Intraperitoneal, intravenous, and peroral routes of administration were investigated without observable TG2 inhibition in either organ (Figure 2A and data not shown). As a control, mass spectrometry was used to verify the presence of the dihydroisoxazole inhibitors at high (∼10 µM) plasma concentrations ([18], data not shown). Finally, in order to confirm that the putrescine incorporation assay was indeed measuring TG2 activity rather than the activity of another transglutaminase, small intestines were harvested from four wild-type and four TG2 knockout mice. The average intestinal transglutaminase activity was 77±8 pmol putrescine/min-mg protein in tissue homogenates from wild-type animals and 4±2 pmol putrescine/min-mg protein in corresponding samples from the TG2 knockout animals.

In order to directly access the small intestine, 2.4 mM of compound **2A** was perfused through the small intestine of an anesthetized rat for 12 minutes. Vehicle perfused sections, proximal and distal to the section in which the inhibitor was perfused, were established as controls. Following perfusion, tissue samples were harvested from each section, frozen in OCT and subsequently cut to 6 µm thickness. TG2 activity was measured *ex vivo* by incubating tissue sections with 5-biotinamidopentylamine (5-BP) in a calcium-rich buffer (thereby activating most or all of the uninhibited TG2). No difference was observed in 5-BP abundance between control- and inhibitor-perfused tissue sections (Figure 2B). Intestinal perfusion of the small molecule amine TG2 substrate 5-BP also did not result in any appreciable signal indicative of *in vivo* TG2 activity (data not shown). To verify that this *ex vivo* TG2 activity assay was indeed specific for TG2 and not another transglutaminase, both transglutaminase activity and TG2 protein were visualized in the sectioned tissue, along with an anti-fibronectin antibody to provide a frame of reference. Nearly perfect overlap was observed for all three stains (Figure 2C).

### Localization, activation and inhibition of TG2 in a tissue wounding model

**Figure 3:**
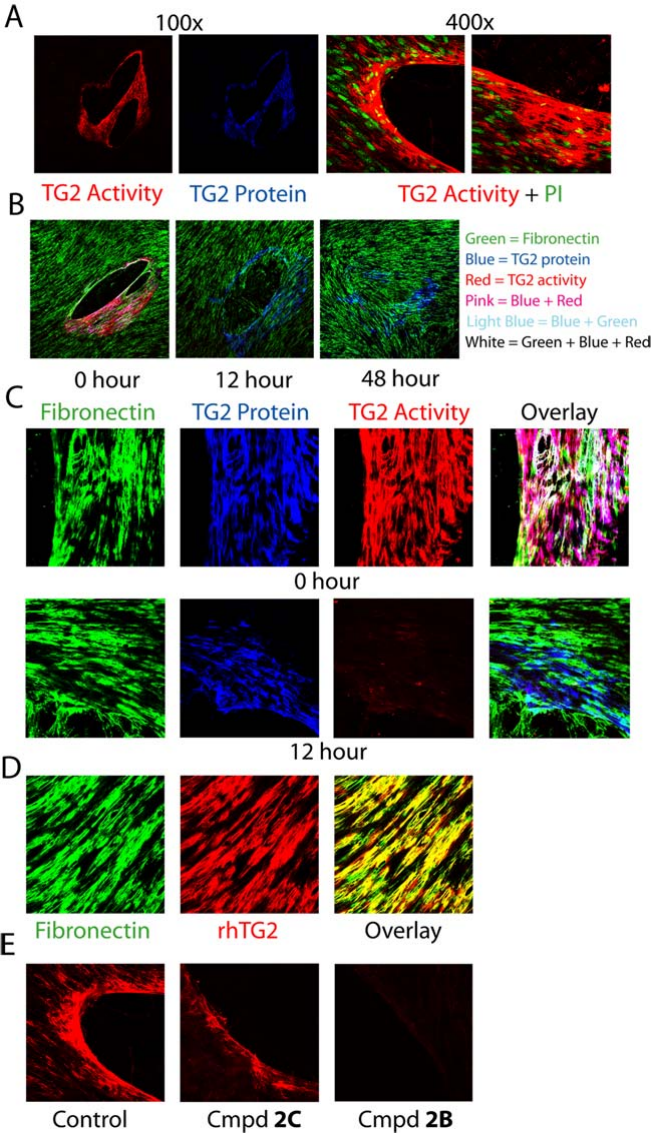
Activation of TG2 in a fibroblast wounding assay. (A) TG2 activity (red) and TG2 protein (blue) were visualized *in situ* after scratching a confluent WI-38 monolayer with a small pipette tip. WI-38 nuclei were visualized with propidium iodide (PI) to illustrate the relative size of a typical scratch compared to an individual cell. No significant TG2 activity was detected in unscratched regions of the WI-38 monolayer. (B) TG2 is enzymatically inactivated within 12 hours of WI-38 wounding despite the persistence of anti-TG2 protein staining. WI-38 monolayers were scratched 48, 12, and 0 hours before adding 5-BP to test for TG2 activity (red). The cells were co-stained for fibronectin (green) and TG2 (blue). (100×) (C) Triple staining of scratched WI-38 monolayers for fibronectin (green), TG2 (blue), and TG2 activity (red) revealed nearly perfect overlap between TG2 and TG2 activity (purple) but only partial overlap with fibronectin (white). This partial overlap of TG2 protein (blue) and fibronectin (green) was not observed 12 hours after wounding. (400×) (D) Addition of exogenous recombinant human TG2 to the monolayers resulted in TG2 deposition onto the WI-38 assembled fibronectin matrix (colocalization in yellow). (E) TG2 inhibitor 2B was able to block *in situ* TG2 activity, whereas the control compound 2C could not block activity.

Wound healing is an established model system for detecting TG2 activity [33]. In this model, catalytically active TG2 is hypothesized to provide mechanical stability to tissues by crosslinking ECM proteins after a wound has been inflicted. In order to study the localization and activity status of TG2 in a simplified cellular model of tissue wounding, WI-38 fibroblast confluent monolayers were scratched with a small pipette tip and analyzed for TG2 protein content and activity by fluorescence microscopy. Although uninjured WI-38 monolayers showed near background enzyme activity levels despite the presence of extracellular TG2 [27], [28], a high density of TG2 protein and activity were visualized around the wounded area (Figure 3A). Comparison of TG2 activity in WI-38 monolayers bearing 20 minute old wounds versus freshly wounded monolayers showed no difference in crosslink formation, suggesting that the region of intense enzymatic TG2 activity surrounding the wound was not due to active export (Figure S1A). In order to gauge how long TG2 protein remains active after wounding, scratches were made 48, 24, 12, and 0 hours before adding the TG2 substrate 5-BP. Interestingly, TG2 activity could only be detected at the 0 hour time-point despite the presence of anti-TG2 antibody staining around the wounded area for all time-points, even the 48 hour time-point with completely healed wounds (Figure 3B–C).

Furthermore, incubation of the fibroblasts with exogenous recombinant human TG2 resulted in exclusive co-localization with the extensive fibronectin ECM network constructed by the cells, whereas TG2 protein and crosslinking activity around the wounded area showed only a partial overlap with fibronectin (Figure 3C–D). Thus, catalytically active TG2 observed after monolayer wounding may be liberated from lysed cells and/or extracellular TG2 that was sequestered in an inactive form before scratching. Propidium iodide nuclear staining of WI-38 cells after wounding confirmed the presence of lysed cells localized exclusively around the wound although the stained nuclei did not always co-localize with TG2 activity (Figure S1B). Scratched monolayers treated with TG2 irreversible inhibitor **2B** completely blocked TG2 activity, whereas incubation with **2C**, an inactive diastereomer of **2B**, was unable to inhibit TG2 activity (Figure 3E).

### Small intestinal TG2 is activated following small intestinal injury with poly(I∶C)

Encouraged by the activation and inhibition of TG2 in the fibroblast wounding model, we evaluated a mouse model of small intestinal wounding to determine whether TG2 would be activated following chemically induced tissue damage. Polyinosinic-polycytidylic acid (poly(I∶C)) is a double-stranded RNA analogue that activates the innate immune pattern recognition receptor, toll-like receptor 3 (TLR3), in the small intestine [26]. Previous work has shown that activation of TLR3 by poly(I∶C) causes human mast cells to detach from fibronectin in the extracellular matrix [34]. Because TG2 is known to be an integrin-binding adhesion co-receptor for fibronectin [27], [28], we chose this small intestinal injury model to determine if small intestinal TG2 could be activated. Mice were given a single intraperitoneal dose of 30 mg/kg poly(I∶C) to induce small intestinal damage. Of this cohort, mice were either intraperitoneally injected with 100 mg/kg 5-BP after 0 hours and 3 hours and sacrificed at 6 hours or injected with 5-BP after 6 hours and 9 hours and sacrificed at 11 hours. Control groups included mice treated with either poly(I∶C) and 2 injections of PBS (poly(I∶C)/PBS), PBS and 2 injections of 5-BP (PBS/5-BP), or PBS and 2 additional injections of PBS (PBS/PBS).

**Figure 4:**
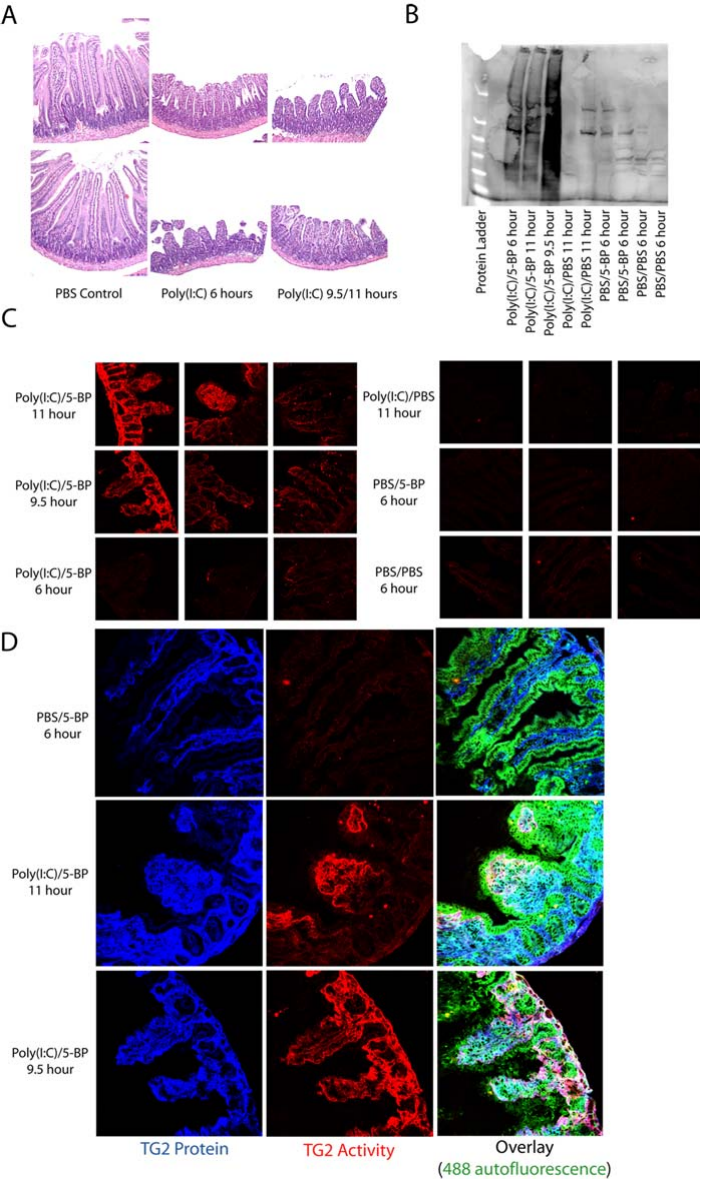
Small intestinal injury by poly(I:C) induces activation of normally latent small intestinal TG2. Mice were injected with 30 mg/kg poly(I:C) at t = 0 to induce injury to the small intestine while controls received vehicle (PBS). Mice were then injected with either 100 mg/kg 5-BP or PBS after 0 hours and 3 hours then sacrificed at 6 hours (labeled “6 hours”) or injected after 6 hours and 9 hours then sacrificed at 11 hours (labeled “11 hours”). One mouse became sick and needed to be sacrificed after 9.5 hours (labeled “9.5 hours”). (A) H&E staining of small intestinal tissues revealed obvious villous atrophy at both the 6 hour and 11 hour time-points following poly(I∶C) injection. (B) Small intestinal tissue lysates were Western blotted using HRP-conjugated streptavidin to detect 5-BP labeled proteins. All mice receiving the combination of poly(I∶C) and 5-BP showed extensive protein labeling while all controls showed background labeling. (C) Immunofluorescent staining of OCT intestinal tissue sections with fluorescent streptavidin revealed the localization of 5-BP labeled proteins in the poly(I∶C)/5-BP mice but showed no labeling in any of the control tissues. (D) Fluorescent streptavidin staining of intestinal tissue from poly(I∶C)/5-BP treated mice showed co-localization with anti-TG2 antibody staining suggesting that the 5-BP labeling was due to TG2 activity.

As shown in Figure 4A, poly(I∶C) rapidly induced villous atrophy within 6 hours of injection and tissue damage was maintained through the 12 hour time-point as reported previously [26]. Moreover, Western blots of small intestinal tissue homogenates using HRP conjugated streptavidin to detect proteins covalently labeled with 5-BP showed extensive protein labeling in the poly(I∶C)/5-BP group of mice but only background labeling in all other groups (Figure 4B). To visualize the tissue localization of 5-BP labeled proteins, OCT tissue sections were stained with fluorescently labeled streptavidin and viewed using fluorescence microscopy. All control tissue sections showed background levels of staining while three out four poly(I∶C)/5-BP mice showed marked increases in TG2 activity (Figure 4C, Figure S2). Moreover, the 5-BP signal in the latter sections overlapped with anti-TG2 staining, suggesting that the 5-BP labeling was due to TG2 enzymatic activity (Figure 4D).

## Discussion

Transglutaminase 2 mediated transamidation and deamidation reactions are thought to contribute to the pathogenesis of a wide range of diseases [17]. However, because TG2 is also believed to contribute to many important biological processes, it is thought that pharmacological inhibition of TG2 may have undesirable side effects. This hypothesis assumes that a fraction of TG2 is constitutively active *in vivo*, and that this activity is important under normal physiological conditions. Yet, data supporting a physiological role for the catalytic activity of TG2 is scarce. In fact, the normal development and normal phenotype of TG2 knockout mice [35], [36], together with the lack of toxicity observed in mice dosed chronically with dihydroisoxazole inhibitors [18], [20], [37] suggest that TG2 activity is unnecessary for survival in a stress-free environment.

An intrinsic difficulty in studying the biology of TG2 enzyme activity is its localization in multiple cellular compartments and association with a variety of protein binding partners. Earlier reports [24], [25] suggest that cytosolic TG2 in healthy cultured cells is catalytically inactive due to the low calcium, high GTP environment inside the cell. In contrast, the enzymatic activity of extracellular TG2 is unclear because, although calcium is abundant, the TG2 is associated with extracellular matrix proteins such as fibronectin and integrins [27], [38]. We therefore sought to investigate the catalytic activity of extracellular TG2 under normal and stressed conditions.

Although TG2 protein and calcium-activated TG2 activity co-localize with fibronectin-bound TG2 in the small intestine (see Figure 2B–C), our data (see Figures 2B, 4C, S2 and S3E) suggests that extracellular TG2 in this organ is typically inactive even though the concentrations of calcium and GTP in the extracellular environment should favor TG2 activity. We therefore sought to verify that TG2 stabilizes and remodels the ECM following tissue damage [1], [39]. Consistent with this hypothesis, abundant TG2 activity was detected around a wound in a scratched fibroblast monolayer while almost no TG2 activity was detected in uninjured regions of the monolayer despite the presence of cell surface TG2 on WI-38 fibroblasts [27], [28]. Because fibroblasts wounded 20 minutes prior to the addition of the 5-BP TG2 substrate showed the same activity profile as fibroblasts immediately wounded in the presence of 5-BP (Figure S1A), this pool of activated TG2 did not appear to be secreted by the wounded fibroblasts. This hypothesis is also supported by the presence of propidium iodide positive cells around the wounded area, which is indicative of loss of plasma membrane integrity during the wounding process (Figure S1B). Therefore, at least a portion of the TG2 activity was caused by the loss of calcium homeostasis in lysed cells. Co-staining with fibronectin indicated only a partial overlap between TG2 protein (or TG2 activity) and fibronectin (see Figures 3B–C). A fraction of the non-fibronectin associated activity presumably came from the activation of TG2 inside partially lysed cells that had lost calcium homeostasis due to plasma membrane disruption but were still able to retain their intracellular protein content. Overall, our data indicates that intracellular TG2 is rapidly activated once a cell loses plasma membrane integrity. Thus, experiments attempting to measure the activity of extracellular TG2 in cell culture require a conditioned media control to account for TG2 released into the media due to cell lysis during the experiment.

Based upon the activation of TG2 in the fibroblast wounding model, we attempted to demonstrate injury-induced TG2 activation *in vivo*. Our initial experiments focused on a methotrexate (MTX) model of small intestinal injury [40], [41]. Although a previous report had shown an increase in TG2 protein expression in the small intestines of MTX-dosed rats [42], we were unable to observe significant changes in TG2 expression in conjunction with dose- or time-dependent damage to the small intestinal mucosa of mice treated with MTX (Figures S3, S4). Moreover, attempts to detect the *in vivo* enzymatic activation of the normally inactive small intestinal TG2 following MTX induced injury were unsuccessful (Figure S3). In contrast, the double-stranded RNA analogue poly(I∶C) resulted in extensive TG2 activation in the mouse small intestine (Figure 4). The latter result is consistent with an earlier observation that poly(I∶C)-mediated activation of TLR3 leads to decreased cell adhesion to the ECM protein fibronectin [34]. It is unclear whether the differences in TG2 activation observed using MTX or poly(I∶C) to induce small intestinal injury are model specific or a result of differences in the time-points taken. In the MTX model, the first group of mice was sacrificed on day 5 whereas in the acute poly(I∶C) injury model all mice were sacrificed within 11 hours of injection. It is possible that TG2 was activated at some point during the first four days following MTX administration, and subsequently inactivated. For example, the WI-38 fibroblast injury model showed that TG2 activity was inactivated within 12 hours after the wound was inflicted, notwithstanding the continued presence of the protein (Figures 3B–C). Therefore, activation of the large but catalytically inactive pool of TG2 in the small intestine following tissue injury appears to be a relatively rapid and short-lived phenomenon.

Toll-like receptors (TLR) are innate immune system receptors that bind to common pathogenic components, such as lipopolysaccharide, cell wall components, or double-stranded RNA, resulting in an outside-in cellular signaling cascade that ultimately causes activation of the immune system. Our data indicates that one potential consequence of the outside-in signal caused by stimulation of a TLR or related receptor may be the generation of an inside-out signal triggering enzymatic activation of extracellular TG2. Recent data indicates that TLR2 and TLR4 expression are upregulated in the intestinal mucosa of treated and untreated celiac sprue patients [43]. Combined with our findings, we speculate that the presence of elevated toll-like receptors in celiac mucosa leads to a constitutively higher level of extracellular TG2 activity. Once activated, TG2 selectively deamidates gluten peptides making them potent antigens and resulting in stimulation of HLA-DQ2 restricted CD4^+^ T cells residing in the intestinal lamina propria [15].

In summary, our data demonstrates that intracellular as well as extracellular TG2 is catalytically inactive under normal physiological conditions, and that physical or certain types of chemical injury can lead to rapid enzymatic activation. Parenthetically we note that our recent X-ray crystal structure of human TG2 bound to an active site inhibitor has revealed that the protein undergoes a large (>100 A) conformation change upon activation into its catalytically active form [44]. Whereas intracellular conditions (low Ca^+2^, high GTP) favor the inactive conformation, we speculate that cell surface TG2 remains inactive due to its association with integrins and its ability to form ternary complexes with cell surface integrin and ECM fibronectin. Physical injury results in release of intracellular TG2, which rapidly assumes an active conformation as a result of GTP dissociation and Ca^+2^ binding. If so, then our data suggests that chemical injury, such as the stimulation of toll-like receptors, triggers release of extracellular TG2 from cell surface integrin and concomitant enzyme activation. Thus, nature appears to have evolved an elaborate mechanism for the post-translational regulation of enzymatic activity of this multifunctional protein in response to diverse physical and chemical stress signals. On one hand, the mammalian body needs a relatively non-specific crosslinking enzyme in virtually every organ in order to facilitate rapid tissue growth and healing. On the other hand, given the extreme stability of an isopeptide linkage, uncontrolled TG2 activity would likely lead to excessive scar tissue formation as well as the formation of neo-epitopes that could trigger an autoimmune response, as in the case of celiac sprue. Thus, the catalytic activity of TG2 must be activated rapidly when needed, and inactivated soon after its task is complete. Perturbation of this delicate balance may be the basis of a variety of seemingly unrelated diseases.

## Methods

### Cell culture and antibodies

WI-38 fibroblasts were grown in minimum essential media (Gibco, 11095) containing L-glutamine, Earle's salts, non-essential amino acids (Gibco, 11140), sodium pyruvate (Gibco, 11360), 10% fetal bovine serum (FBS) (Atlanta Biologicals), and 100 U/ml penicillin+100 µg/ml streptomycin (Gibco, 15140). MDA-MB-231 cells were grown in RPMI-1640 with L-glutamine (Gibco, 11875), 10% FBS (Atlanta Biologicals), and 100 U/ml penicillin+100 ug/ml streptomycin (PS). Cells were typically grown to 80–90% confluency before treating with trypsin-EDTA (Gibco, 15400) and splitting. All cells were grown in a humidified incubator at 37°C, 5% CO_2_. The antibodies used were a rabbit polyclonal anti-TG2 antibody (Lab Vision Corp., RB-060), CUB7402/TG100 mouse anti-TG2 antibody mix (Lab Vision Corp., MS-300), rabbit anti-fibronectin (Sigma, F3648), Alexa fluor 488 goat anti-rabbit antibody (Invitrogen, A-11008), Alexa fluor 568 goat anti-mouse antibody (Invitrogen, A-11004), Alexa fluor 647 labeled streptavidin (Invitrogen, S21374), and Alexa fluor 514 labeled streptavidin (Invitrogen).

### Transglutaminase inhibitor synthesis

The TG2 inhibitors used in this study are summarized in Figure 1A. 3-bromo-4,5-dihydroisoxazole inhibitors were synthesized according to previously published procedures [18], [19]. New TG2 inhibitor (compound **1**) was prepared as follows.

**(** ***S*** **)-** ***N*** **-(((** ***S*** **)-3-bromo-4,5-dihydroisoxazol-5-yl)methyl)-2-(1-(dimethylamino) naphthalene-5-sulfonamido)-3-(1** ***H*** **-indol-3-yl)propanamide (1)** 59.6 mg (0.111 mmol) N-dansyl-L-tryptophan was dissolved in 1.11 mL neat DMF (0.1M), and placed in a water/ice bath. 21.8 mg of 7-amino-3-bromo,4-,5-dihydroisoxazole (0.122 mmol, 1.1 equiv) were added to the reaction mixture followed by 16.5 mg (0.122 mmol, 1.1 equiv) of hydroxybenzotriazole hydrate, and 23.4 mg of EDCI (0.122 mmol, 1.1 equiv). The reaction was allowed to run for 12 hrs, was followed by an aqueous workup with 1M phosphate buffer at pH 3, extraction with EtOAc (5×5ml), drying over sodium sulfate, concentration under reduced pressure and purification by prep. TLC in 4:1 dichloromethane: acetone (yield 71.8%). ^1^H NMR (500MHz, CDCl_3_): δ 1.24–1.27 (dd, 1H, J = 7.0,7.0), 1.98–2.00 (m, 1H, J = 5.0), 2.022 (s, 1H), 2.22 (s, 1H), 2.65–2.70 (dd, 1H, J = 8.0, 17.5), 2.91–2.93 (dd, 1H, J = 0.5, 17.5), 2.96–3.06 (m, 3H), 3.10–3.16 (ddd, 1H, J = 5.0, 6.0, 14.0), 3.90–3.94 (ddd, 1H, J = 6.0, 7.5, 14.0), 4.086–4.128 (ddd, 1H, J = 7.0, 14.0, 7.0), 4.39–4.45 (m, 1H), 6.18–6.19 (d, 1H, J = 7.5), 6.67–6.69 (dd, 1H, J = 5.5, 5.5), 6.91–6.92 (d, 1H, J = 2.5), 6.93–6.97 (ddd, 1H, J = 1.0, 7.0, 8.0), 7.06–7.10 (ddd, 1H, J = 1.0, 7.0, 8.0) 7.24–7.27 (m, 2H), 7.27–7.29 (ddd, 1H, J = 1.0, 2.0, 6.0), 7.43–7.57 (ddd, 2H, J = 6.5, 1.5, 56.0), 8.07–8.09 (dd, 1H, J = 1.0, 7.0), 8.15–8.17 (ddd, 1H, J = 0.5, 0.5, 8.5), 8.45–8.47 (ddd, 1H, J = 1.0, 1.0, 8.5), 8.91 (bs, 1H). [α]^22^_D_ (c = 0.5, ACN) = +20.096.

### SDS PAGE and Western Blotting

Samples were diluted 1∶1 with 2× Laemmli sample buffer containing β-mercaptoethanol, and boiled for 10 mins. Samples were applied to 4–12% or 4–15% SDS PAGE gels (Bio-Rad, Invitrogen). For Western blotting, proteins were transferred onto a nitrocellulose membrane at 80 volts for 1 hour in transfer buffer (2.42 g Tris, 11.2 g glycine to 800 ml plus 200 ml methanol). Membranes were blocked with 2% non-fat dry milk in PBS (blocking buffer) for 30 minutes at room temp. Horseradish peroxidase conjugated neutravidin (Invitrogen) was incubated with the nitrocellulose membrane overnight at 4°C in blocking buffer. The substrate ELC plus (GE Life Sciences) was used to visualize staining according to the manufacturers protocol. The membrane was scanned using a Typhoon fluorescence imager (GE Life Sciences).

### Cell culture inhibition experiments

To quantify TG2 inhibition in cultured cells, cells (MDA-MB-231 and WI-38) were grown to near confluence. Dihydroisoxazole inhibitor **1** was diluted to its indicated concentration in culture media, and vehicle (0.5% DMSO) was added to the media of control cells. Cells were incubated with the inhibitor for the indicated amount of time at 37°C, 5% CO_2_ before being washed with PBS (pre-warmed to 37°C) for 15 mins in the incubator to allow any free inhibitor to diffuse out from cells. MDA-MB-231 cells were detached with 10 mM EDTA in PBS at 37°C to avoid proteolysis of extracellular TG2. However, trypsin-EDTA detachment was necessary for the ECM-rich WI-38 cells. Cell pellets were resuspended to approximately 7*10^6^ cells/ml in 50 mM mannitol, 2 mM tris, 1 mM EDTA (MTE) buffer. Cells were lysed by sonicating 5× for 8 sec. each using a microtip sonicator. The cell lysate was divided into four 80 µl aliquots and either 1% DMSO, 1% DMSO+5 mM CaCl_2_, 1% DMSO+100 uM **1**, or 1% DMSO+100 uM **1**+5 mM CaCl_2_ was added to the aliquots and allowed to incubate for 30 mins at room temp. Putrescine incorporation in a calcium-rich buffer was then used to measure the amount of TG2 activity in the lysates.

### In vivo inhibition experiments

Dihydroisoxazole inhibitors were dissolved in solutions ranging from 50%–100% DMSO in PBS. Controls received vehicle only. For experiments in mice, inhibitor was injected either intraperitoneally (IP) or retro-orbitally (IV) at 100 µl per 20 g mouse weight. Mice were sacrificed using CO_2_ inhalation one hr after injection followed by harvesting of the small intestine and liver. The liver was immediately frozen in liquid nitrogen. In some experiments, the small intestine lumen was rinsed with PBS using a needle and syringe. The small intestine was cut longitudinally, and the mucosa was scraped using microscope slides. Intestinal mucosa was wrapped in parafilm and frozen on dry ice. In some experiments, blood was collected by tail bleeding and immediately frozen in liquid nitrogen. Transglutaminase 2 knockout mice and wild-type controls were described previously [45].

Intestinal tissue was homogenized to 20% wt/wt and livers to 10% wt/wt in 50 mM mannitol, 2 mM Tris HCl, 1 mM EDTA, pH 7.4 (MTE) solution using a Wheaton glass dounce homogenizer. Samples were sonicated 2× for 8 sec. with a microtip sonicator. The resulting lysate was used in the putrescine incorporation assay.

### Putrescine incorporation assay

Incorporation of putrescine into N,N-dimethyl casein was measured as described previously [18]. The dimethyl casein substrate buffer contained 10 µM [1,4-^14^C] putrescine and 50 µM unlabeled putrescine, and the reaction was initiated by adding 1 volume of lysate to 4 volumes of calcium-rich substrate buffer. For cell culture inhibition experiments, putrescine incorporation was measured only at 0 and 10 minutes since the presence of dihydroisoxazole inhibitors in some of the samples led to a time-dependent inhibition of TG2 activity that was more visible at later timepoints.

### Rat small intestinal perfusion

Rat intestinal perfusion experiments were performed as described previously [46]. Vehicle (1% N-lauroyl sarcosine for **2A** perfusions and 0.9% NaCl for 5-biotinamidopentylamine (5-BP) perfusions) was perfused through 20 cm of the rat jejunum for 15 mins at a flow rate of 24 ml/hr. Then, the middle 10 cm section of the pre-perfused jejunum was perfused with the compound of interest (2.4 mM **2A** or 500 µM 5-BP) for 15–20 minutes at 24 ml/hr. The animal was sacrificed and tissue sections from the control and compound perfused sections were frozen in OCT on dry ice for subsequent tissue sectioning experiments.

### In situ TG2 activity assay and tissue staining

*In situ* TG2 activity was measured in rodent tissue sections according to a previously published protocol [22] with the following modifications. The 5-biotinamidopentylamine (Pierce, 21345) substrate was incubated with the tissue section for 1 hr at room temp. instead of 2 hours and, in some experiments, the concentration of 5-BP was titrated down to 5 µM. Alexa fluor 647 conjugated streptavidin (1∶100 in blocking buffer) was used to detect 5-BP crosslinked to tissue sections, and Alexa fluor 488 anti-rabbit IgG and Alexa fluor 568 anti-mouse IgG secondary antibodies (1:1000 in blocking buffer) were used to detect primary antibody staining. The slides were mounted using VECTASHIELD mounting medium containing DAPI (Vector Labs). It should be noted that 5-BP was incubated with the tissue section in a buffer containing 5 mM CaCl_2_. Thus, the *in situ* TG2 activity signal is not necessarily indicative of true *in vivo* TG2 activity as demonstrated in the Results section.

### WI-38 fibroblast wound model

The WI-38 fibroblast scratch model was based upon a modification of a previously published protocol [33]. WI-38 fibroblasts were plated in an 8-well chamber glass slide at 10^5^ cells/well and grown for 7–10 days with a media change every 2 days. Small scratches were made in the monolayer with a 0.1–10 µl pipette tip, and the cells were incubated with 100–500 µM 5-BP for 30–60 mins at 37°C. The cells were washed 3× with PBS then fixed with −20°C methanol for 10 mins. The cells were washed with PBS 2× for 5 mins, blocked with 1% BSA in PBS for 5 mins at room temp, and then washed 2× using PBS. Alexa fluor 647 labeled streptavidin was diluted 1:500 in blocking buffer and incubated with the cells for 30–60 mins at room temp. The cells were washed 4× with PBS and 300 µl PBS was added onto the cells before viewing with a confocal laser scanning microscope. For propidium iodide (PI) staining, a 1 mg/ml stock solution of PI in PBS was diluted 1∶1000 in PBS and incubated at room temp with cells for 5 mins. Cells were washed 3× and visualized using microscopy.

To determine whether the released TG2 was being actively exported or simply leaking out, the rate of extracellular TG2 accumulation was measured. Scratches were made to monolayers, and the cells were incubated at 37°C for 20 mins in normal media. Then, 500 µM 5-BP was added to these previously scratched wells and also to unscratched wells, which were then immediately scratched. After the indicated time-points, the 5-BP was washed off 3× with PBS. The fixation and staining protocol was the same as described above.

For propidium iodide staining of live cells, stock PI was diluted 1∶1000 in PBS and incubated with live cells for 5 mins at room temp. The monolayer was washed 3× with PBS and the cells were fixed with 4% paraformaldehyde for 10 mins at room temp. The cells were washed 3× with PBS and viewed under the microscope.

For TG2 inhibitor experiments, the inhibitors were diluted into culture medium at 100 µM and incubated with the cells for 20 mins after scratching. Then, 100 µM 5-BP was added to wells and co-incubated with the inhibitors for 30 mins before fixing and staining as described above.

### Poly(I:C) induced small intestinal damage in mice

C57BL/6 mice (8 weeks old) were IP injected with 30 mg/kg poly(I:C) (Sigma, P1530) dissolved in PBS to injure the small intestine as described previously [26]. Controls were given vehicle (PBS only). 5-BP was dissolved to 27.8 mg/ml in water and diluted to 25 mg/ml using 10× PBS. Mice were IP injected twice with either 100 mg/kg 5-BP or PBS at three hr intervals. For mice sacrificed after 6 hrs, 5-BP or PBS was injected at 0 and 3 hrs. For mice sacrificed after 11 hrs (or 9.5 hrs for one mouse), 5-BP or PBS was injected at 6 and 9 hrs. Therefore, 5-BP/PBS was injected between 5–6 and 2–3 hours before sacrifice for both the 6 hour and 11 hour timepoints. The lumens of isolated small intestines were rinsed with PBS using a needle and syringe, and small pieces of tissue were formalin-fixed for hematoxylin and eosin (H&E) staining or frozen in OCT for subsequent immunofluorescence staining. The intestine was cut longitudinally, and the intestinal mucosa was scraped using microscope slides, wrapped in parafilm, and frozen on dry ice. Upon thawing, tissue was homogenized with a Wheaton dounce homogenizer in 50 mM mannitol, 2 mM tris, 10 mM EDTA, pH = 7.4, sonicated 2X, and diluted in Laemmli sample buffer for SDS PAGE and Western blotting. HRP conjugated neutravidin (Invitrogen, A-2664) and ECL plus substrate (GE Biosciences) were used to visualize biotinylated proteins. The four groups of mice (poly(I:C)/5-BP, poly(I:C)/PBS, PBS/5-BP, PBS/PBS) were analyzed in duplicate using one male and one female mouse.

### Methotrexate induced small intestinal damage in mice

Methotrexate (Sigma, 06563) was dissolved in 33% 0.1 M NaOH+66% PBS with a small amount of 2 M NaOH necessary to completely dissolve the compound to 12.5 mg/ml. Mice were orally gavaged with 50 mg/kg methotrexate or PBS daily for the indicated number of days. Animal body weight was monitored daily until the time of sacrifice. Animals were sacrificed using CO_2_ inhalation, and small intestinal tissue was collected, rinsed with PBS using a needle and syringe, and cut longitudinally. Intestinal mucosa was scraped using microscope slides, wrapped in parafilm, and frozen on dry ice. Small cross-sectional tissue pieces were either fixed in formalin or frozen in OCT freezing medium. C57/BL6J mice were used in the first two MTX experiments and FVB mice were used in the FC/5-BP MTX experiments.

Fluorescein cadaverine (FC) (Fisher, PR-P1911) was dissolved in 0.5 M NaOH to 69 mM and further diluted to 14 mM in PBS. The FC concentration was calculated from its absorbance at 495 nm, using 80,500 cm^−1^M^−1^ as the extinction coefficient. Mice were injected IP with three 30 mg/kg FC doses at 29, 17, and 5 hrs before sacrifice. 5-BP was dissolved to 50 mM in water and diluted to 45 mM using 10× PBS. Mice were IP injected with two 120 mg/kg 5-BP doses at 17 and 5 hrs before sacrifice. Control mice were injected with an equivalent volume of PBS. Pharmacokinetic data using a 5 mg/kg dose of FC gave a serum half-life of around 25 mins and peak serum concentration of 15 mM (data not shown). Two mice were injected with 150 mg/kg monodansylcadaverine, a widely used amine substrate of TG2, but it proved to be toxic, leading to animal death within 20 mins.

## Supporting Information

Figure S1 Enzymatically active TG2 is not exported from WI-38 cells near the wound. (A) Small puncture wounds were made to WI-38 fibroblast monolayers either 20 minutes prior to the addition of 5-BP or in the presence of 5-BP. The reaction was allowed to proceed for the indicated time before the monolayers were washed free of 5-BP and fixed/permeabilized with methanol. The localization and intensity of TG2 activity around the 20 minute old wound and fresh wound were nearly identical indicating that the TG2 activity was not due to TG2 actively exported by cells proximal to the wound. (B) Co-staining of wounded WI-38 monolayers for TG2 activity (red) and propidium iodide (PI, a dead cell nuclear stain, blue) showed significant cell lysis around the wounded area, although the TG2 activity staining and PI staining did not always perfectly overlap (co-localization is pink).
(6.96 MB TIF)

Figure S2 Small intestinal injury by poly(I:C) induces activation of normally latent small intestinal TG2. Mouse small intestinal OCT sections from poly(I:C) treated mice and controls were stained with Alexa fluor 514 labeled streptavidin. Representative fluorescent microscopy images from each poly(I:C)/5-BP treated mouse are shown. Images from two control mice are also displayed to show background staining levels.
(2.91 MB TIF)

Figure S3 Methotrexate-induced small intestinal damage does not result in up-regulation or activation of TG2. (A) Mice were perorally dosed daily with between 1–5 doses of MTX as indicated on the graph. On day 7, all mice were sacrificed. Although H&E staining of the tissue revealed dose-dependent damage, there was no apparent change in TG2 expression levels as judged by the putrescine incorporation assay. (B) Mice were given either 3 peroral doses of MTX or vehicle (PBS) daily between days 0–2. Body weight was monitored over the duration of the experiment and plotted as percent body weight based upon day 0 weights. ____(green) Mice given FC; ____(red) Mice given 5-BP; ____(blue) Mice given vehicle (PBS) (C) Formalin fixed small intestinal tissue sections from vehicle treated and MTX treated mice sacrificed on days 5, 8, and 9 were stained with H&E to verify the small intestinal damage caused by MTX. (100X) (D) Small intestinal tissue lysate from vehicle treated and FC treated mice were separated by SDS PAGE and subsequently scanned for FC fluorescence. Although free FC was still present in the tissues, all proteins labeled with FC were also labeled in the lysate from FC injected TG2 knockout mice. Labels indicate the dosing schemes, and TG2 knockout and wild-type mice injected with FC are denoted KO and WT, respectively. (E) In situ TG2 activity could be detected in vehicle treated mouse OCT tissue sections by incubating the section with 5 µM 5-BP in a calcium containing buffer at room temperature for 1 hour. However, no in situ staining representative of in vivo TG2 activity could be detected in two MTX treated mice dosed with 2 intraperitoneal injections of 120 mg/kg 5-BP.
(6.73 MB TIF)

Figure S4 Small intestinal TG2 protein content is not upregulated during methotrexate-induced small intestinal wounding. Mice were perorally dosed on two consecutive days with methotrexate to cause small intestinal damage. Two mice were sacrificed each day over the course of ten days, and their small intestinal mucosa was harvested. Putrescine incorporation was used to quantify the amount of TG2 protein in the intestine. No significant changes in protein expression levels were observed despite intestinal damage.
(0.74 MB TIF)
