# Supplementary figures and images for: Extracellular Transglutaminase 2 Is Catalytically Inactive, but Is Transiently Activated upon Tissue Injury
Source: PLoS One. 2008 Mar 26;3(3):e1861. doi: 10.1371/journal.pone.0001861 (PMC2267210; doi:10.1371/journal.pone.0001861)

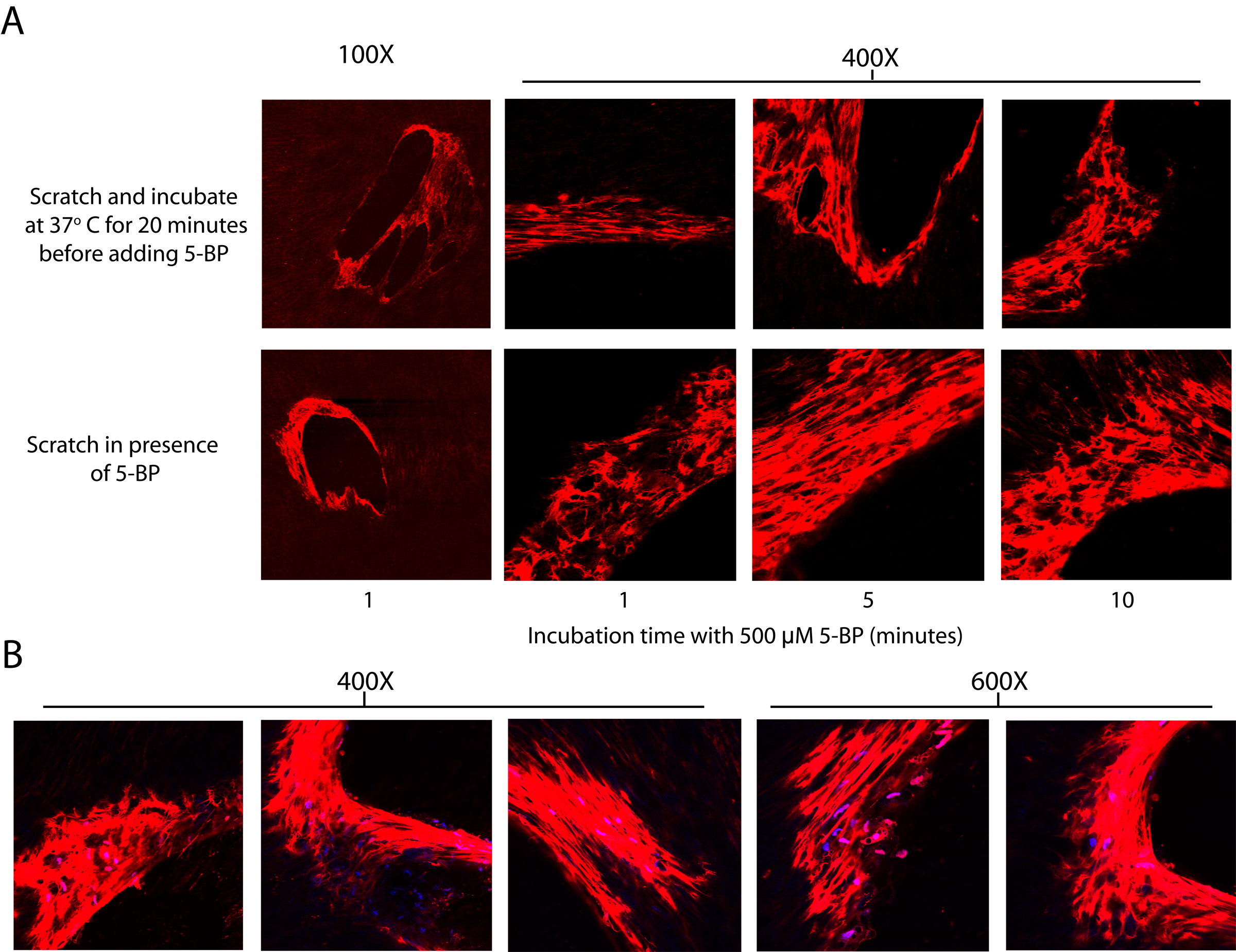

Supplement: Figure S1 — Enzymatically active TG2 is not exported from WI-38 cells near the wound. (A) Small puncture wounds were made to WI-38 fibroblast monolayers either 20 minutes prior to the addition of 5-BP or in the presence of 5-BP. The reaction was allowed to proceed for the indicated time before the monolayers were washed free of 5-BP and fixed/permeabilized with methanol. The localization and intensity of TG2 activity around the 20 minute old wound and fresh wound were nearly identical indicating that the TG2 activity was not due to TG2 actively exported by cells proximal to the wound. (B) Co-staining of wounded WI-38 monolayers for TG2 activity (red) and propidium iodide (PI, a dead cell nuclear stain, blue) showed significant cell lysis around the wounded area, although the TG2 activity staining and PI staining did not always perfectly overlap (co-localization is pink). (6.96 MB TIF) [file pone.0001861.s001.tif]

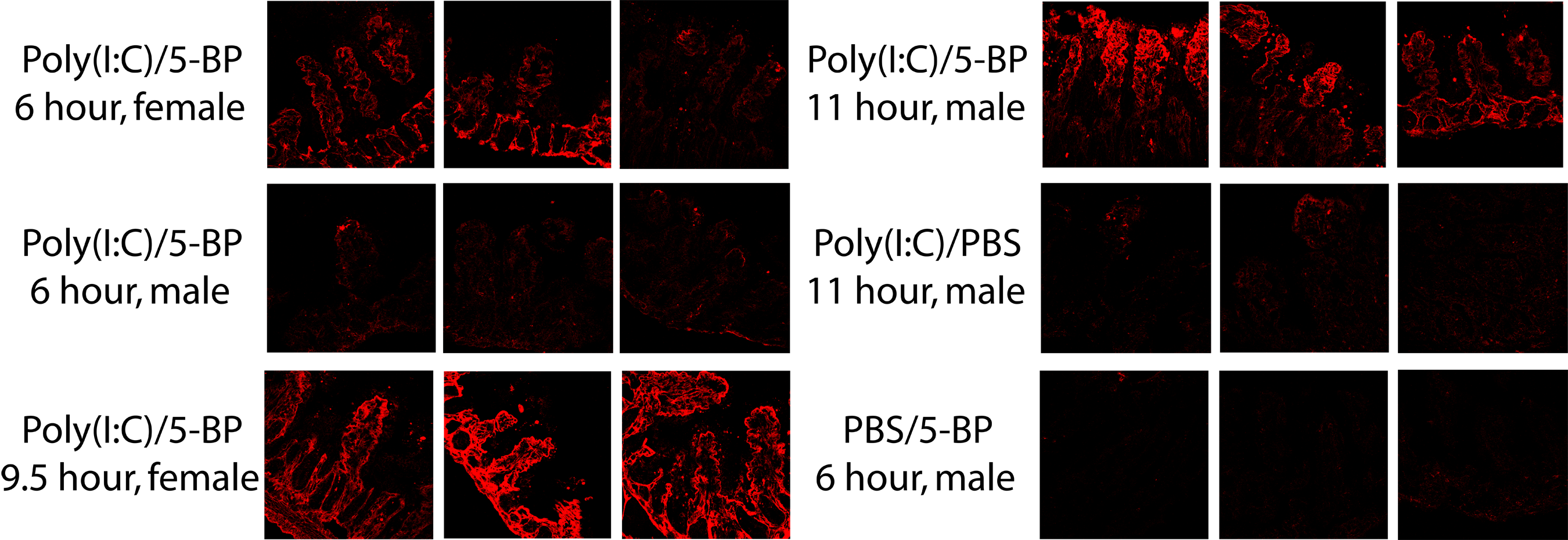

Supplement: Figure S2 — Small intestinal injury by poly(I:C) induces activation of normally latent small intestinal TG2. Mouse small intestinal OCT sections from poly(I:C) treated mice and controls were stained with Alexa fluor 514 labeled streptavidin. Representative fluorescent microscopy images from each poly(I:C)/5-BP treated mouse are shown. Images from two control mice are also displayed to show background staining levels. (2.91 MB TIF) [file pone.0001861.s002.tif]

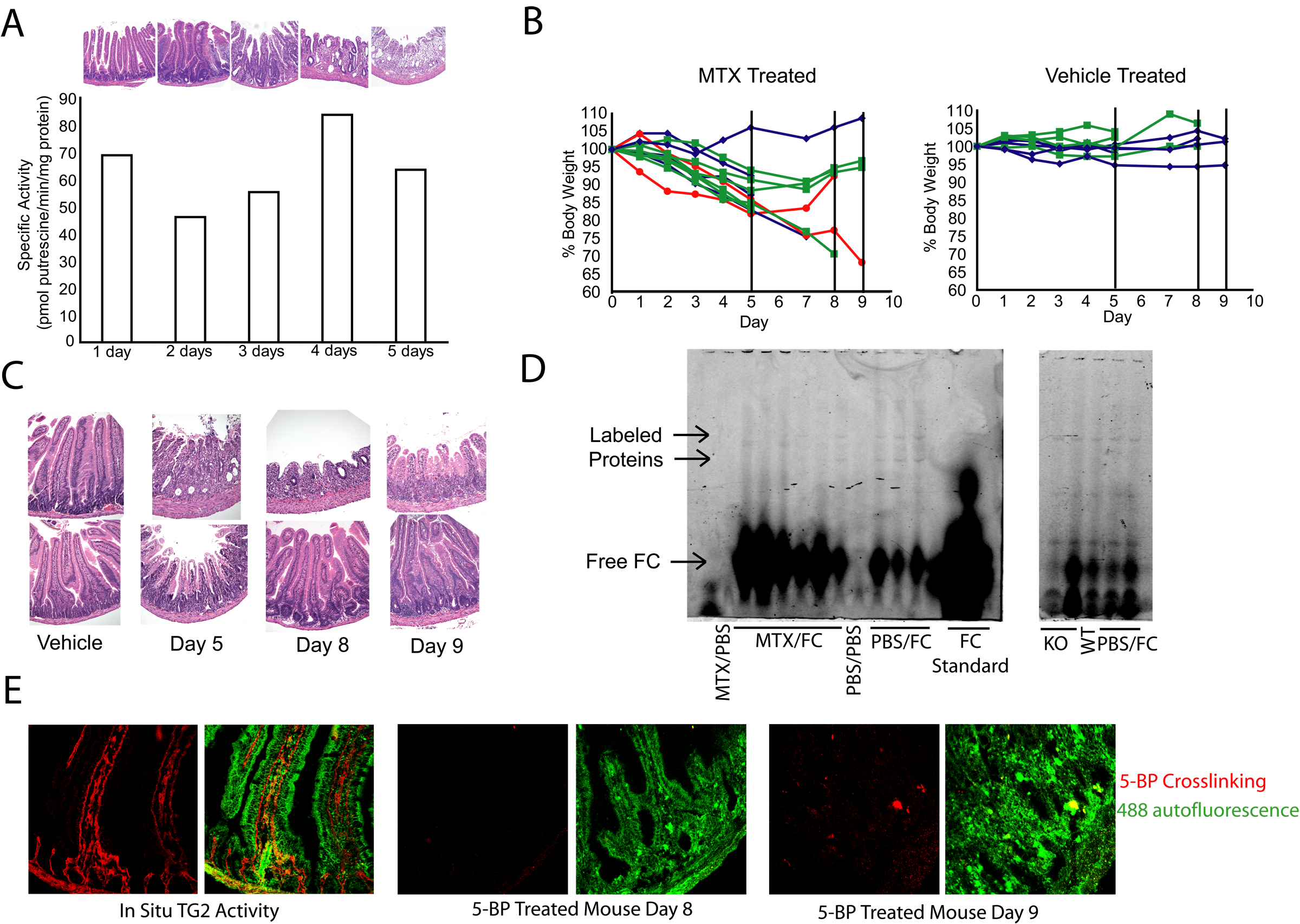

Supplement: Figure S3 — Methotrexate-induced small intestinal damage does not result in up-regulation or activation of TG2. (A) Mice were perorally dosed daily with between 1–5 doses of MTX as indicated on the graph. On day 7, all mice were sacrificed. Although H&E staining of the tissue revealed dose-dependent damage, there was no apparent change in TG2 expression levels as judged by the putrescine incorporation assay. (B) Mice were given either 3 peroral doses of MTX or vehicle (PBS) daily between days 0–2. Body weight was monitored over the duration of the experiment and plotted as percent body weight based upon day 0 weights. ____(green) Mice given FC; ____(red) Mice given 5-BP; ____(blue) Mice given vehicle (PBS) (C) Formalin fixed small intestinal tissue sections from vehicle treated and MTX treated mice sacrificed on days 5, 8, and 9 were stained with H&E to verify the small intestinal damage caused by MTX. (100X) (D) Small intestinal tissue lysate from vehicle treated and FC treated mice were separated by SDS PAGE and subsequently scanned for FC fluorescence. Although free FC was still present in the tissues, all proteins labeled with FC were also labeled in the lysate from FC injected TG2 knockout mice. Labels indicate the dosing schemes, and TG2 knockout and wild-type mice injected with FC are denoted KO and WT, respectively. (E) In situ TG2 activity could be detected in vehicle treated mouse OCT tissue sections by incubating the section with 5 µM 5-BP in a calcium containing buffer at room temperature for 1 hour. However, no in situ staining representative of in vivo TG2 activity could be detected in two MTX treated mice dosed with 2 intraperitoneal injections of 120 mg/kg 5-BP. (6.73 MB TIF) [file pone.0001861.s003.tif]

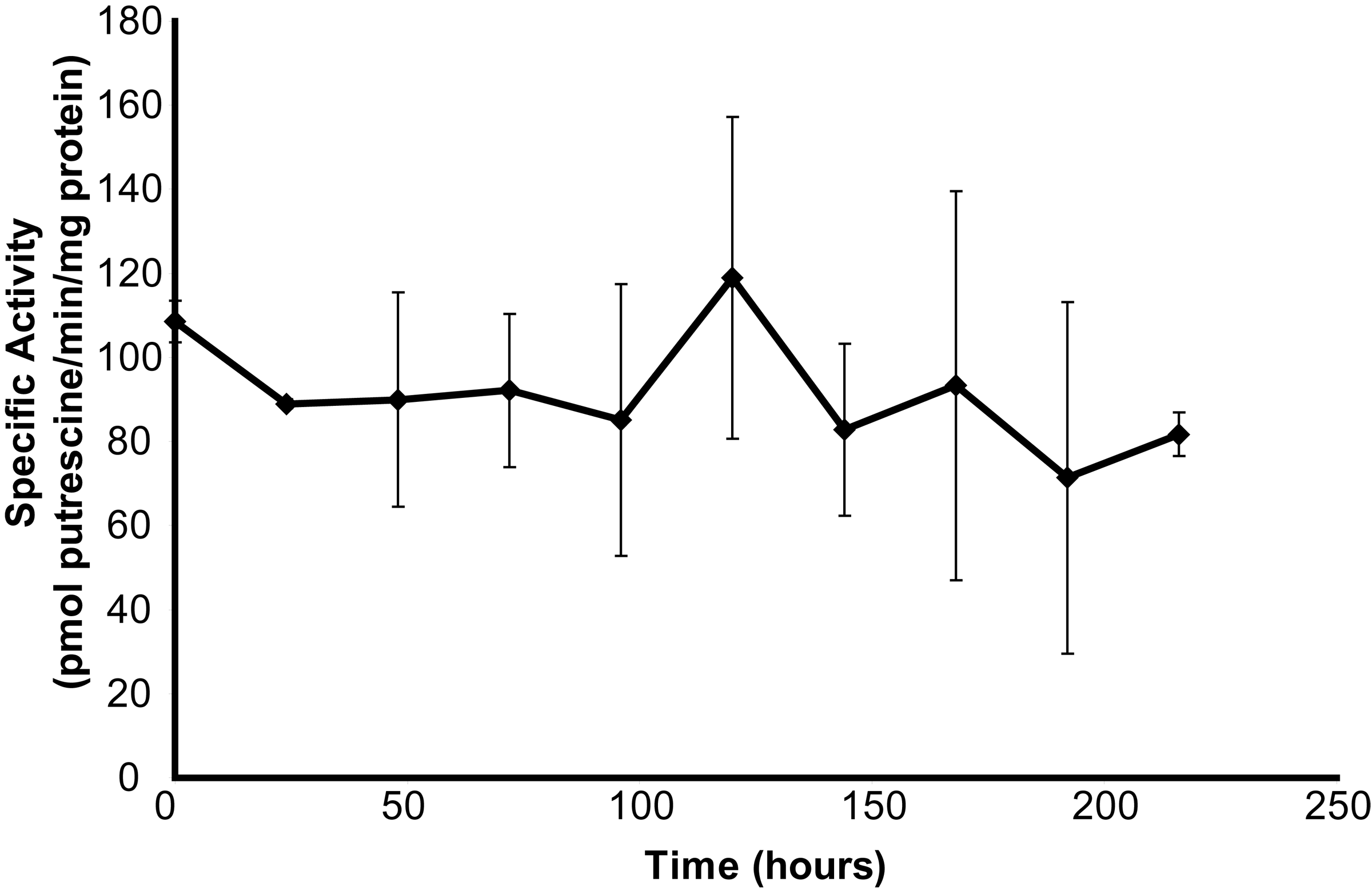

Supplement: Figure S4 — Small intestinal TG2 protein content is not upregulated during methotrexate-induced small intestinal wounding. Mice were perorally dosed on two consecutive days with methotrexate to cause small intestinal damage. Two mice were sacrificed each day over the course of ten days, and their small intestinal mucosa was harvested. Putrescine incorporation was used to quantify the amount of TG2 protein in the intestine. No significant changes in protein expression levels were observed despite intestinal damage. (0.74 MB TIF) [file pone.0001861.s004.tif]
